# Supplementary material for: Characterizing DNA methylation signatures of retinoblastoma using aqueous humor liquid biopsy
Source: Nat Commun. 2022 Sep 21;13:5523. doi: 10.1038/s41467-022-33248-2 (PMC9492718; doi:10.1038/s41467-022-33248-2)
Supplement: Supplementary file 1 — Supplementary Information [file 41467_2022_33248_MOESM1_ESM.pdf]

Supplementary Information for

Characterizing DNA methylation signatures of retinoblastoma using the aqueous humor liquid biopsy: moving beyond genomics

Short Title: DNA methylation signature of retinoblastoma in liquid biopsy

Hong-Tao Li[1#], Liya Xu[2#], Daniel J. Weisenberger[3,4], Meng Li[5], Wanding Zhou[6], Chen-Ching Peng[2], Kevin Stachelek[2], David Cobrinik[2,3,4,7], Gangning Liang[1,4*] and Jesse L. Berry[2,4,7*]

[1]Department of Urology, University of Southern California, Norris Comprehensive Cancer Center, Los Angeles, CA 90033 USA

[2]Children's Hospital Los Angeles Vision Center & USC Roski Eye Institute, Keck School of Medicine, University of Southern California, Los Angeles, CA 90027

[3]Department of Biochemistry and Molecular Medicine, University of Southern California, Norris Comprehensive Cancer Center, Los Angeles, CA 90033 USA

[4]Norris Comprehensive Cancer Center, Keck School of Medicine, University of Southern California, Los Angeles, CA

[5]Norris Medical Library, Universirty of Southern California, Los Angeles, CA 90033

[6]University of Pennsylvania, Children's Hospital of Philadelphia, Philadelphia, PA 19104

[7]The Saban Research Institute, Children's Hospital Los Angeles, Los Angeles, CA

[#] Contribute equally to the manuscript
* To whom correspondence should be addressed:
jesse.berry@med.usc.edu, Phone: 323-442-6578
gliang@usc.edu, Phone: 323-865-0470

A

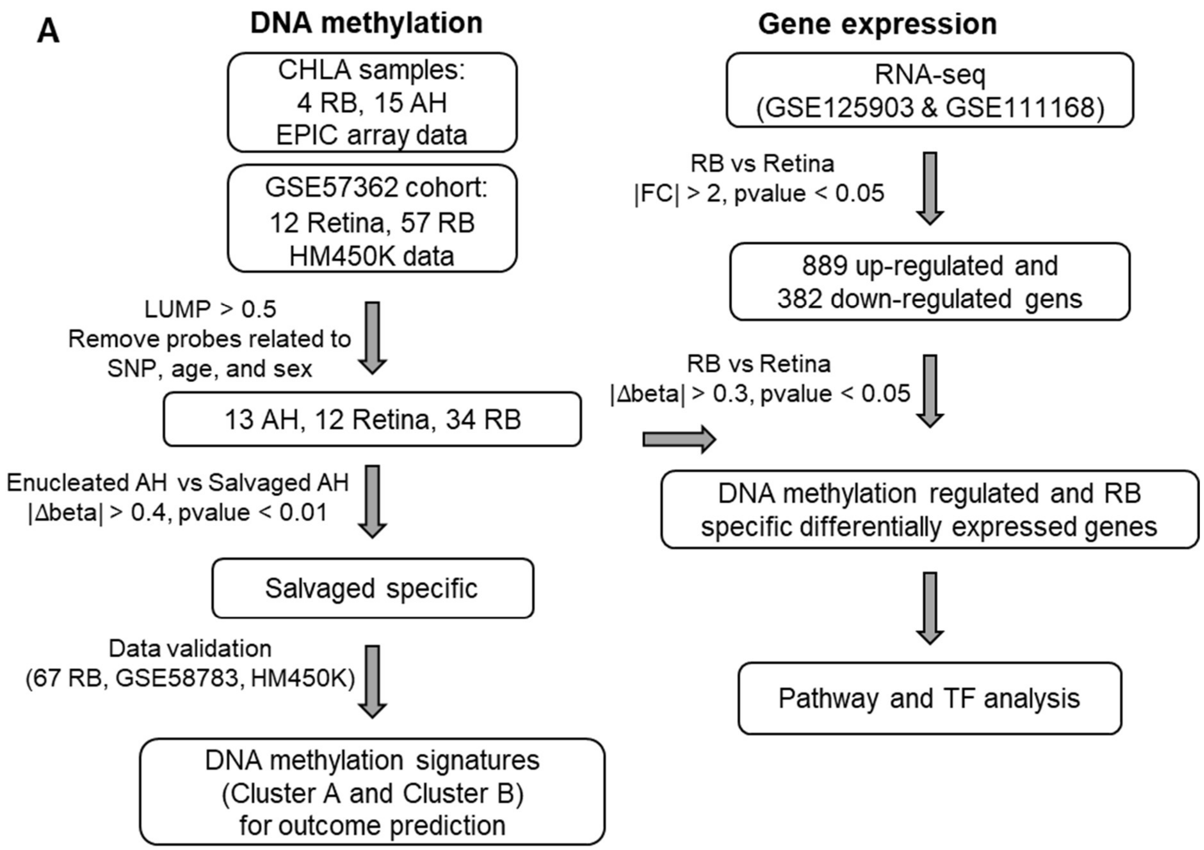

B

Source	Sample Type	Sample Name	Outcome	Sample Number	Clusters (A/B)
**CHLA**	Primary Tumor	RB_CHLA	Primary enucleated	4	0/4
	Aqueous Humor (cfDNA)	AH_CHLA	Primary enucleated	4	0/4
			Secondary enucleated	6	0/4
			Salvaged	5	4/0
	RB Blood	Blood_CHLA		2	
**GSE57362**	Retina	Retina		12	
	Primary Tumor	RB_SR	Enucleated	30	4/9
	RB Blood	Blood_SR		27	
**GSE58783**	Primary Tumor	RB_NC	Enucleated	67	19/24

Supplementary Figure S1. Datasets and analysis outline.

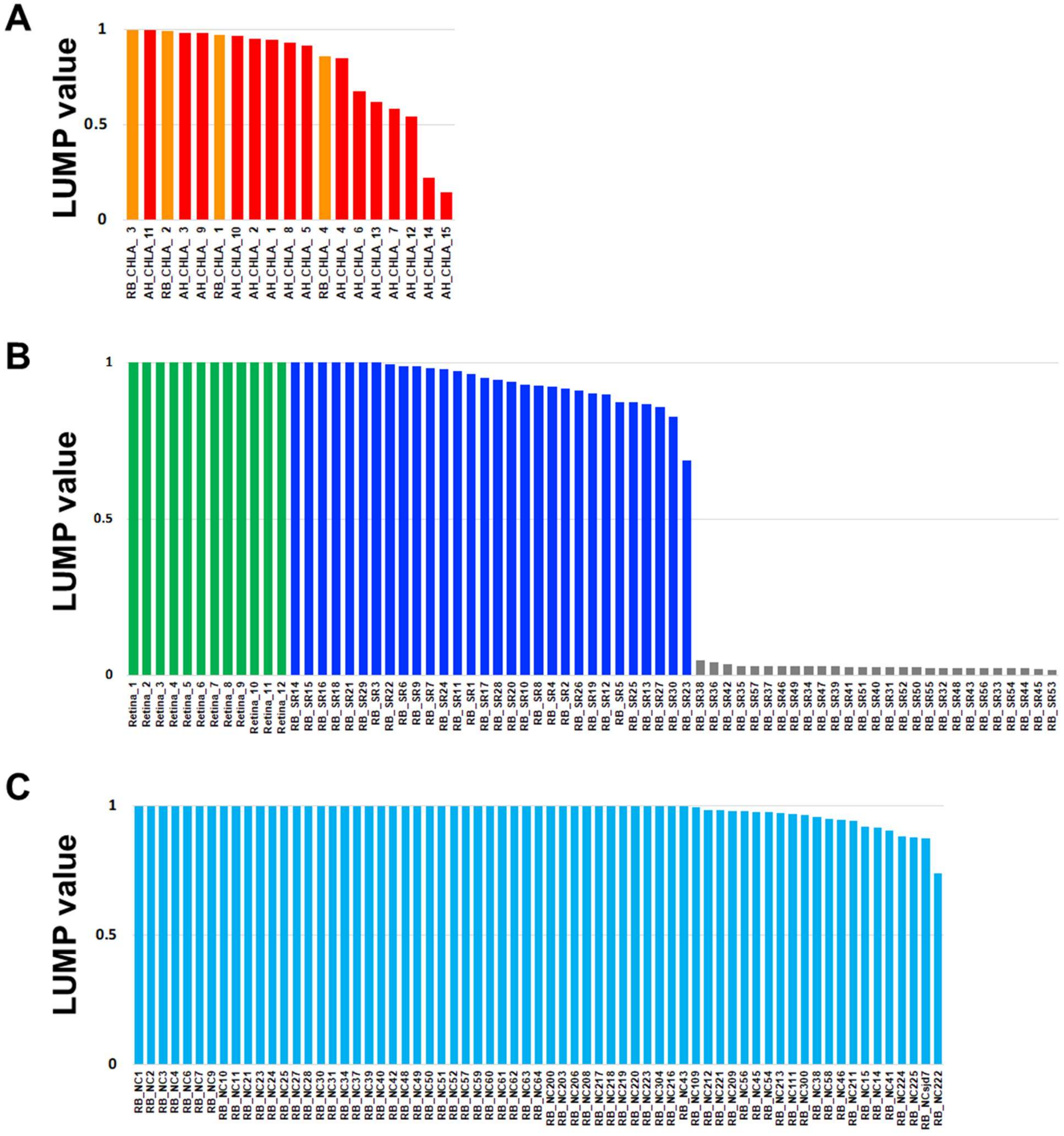

Supplementary Figure S2. LUMP assay filtering the samples purity on the RB_CHLA (orange) and AH_CHLA (red) for this study (A), retina (green) and RB (blue) from GSE57362 (B), and RB (light blue) from GSE58783 (C). Source data are provided as a Source Data file.

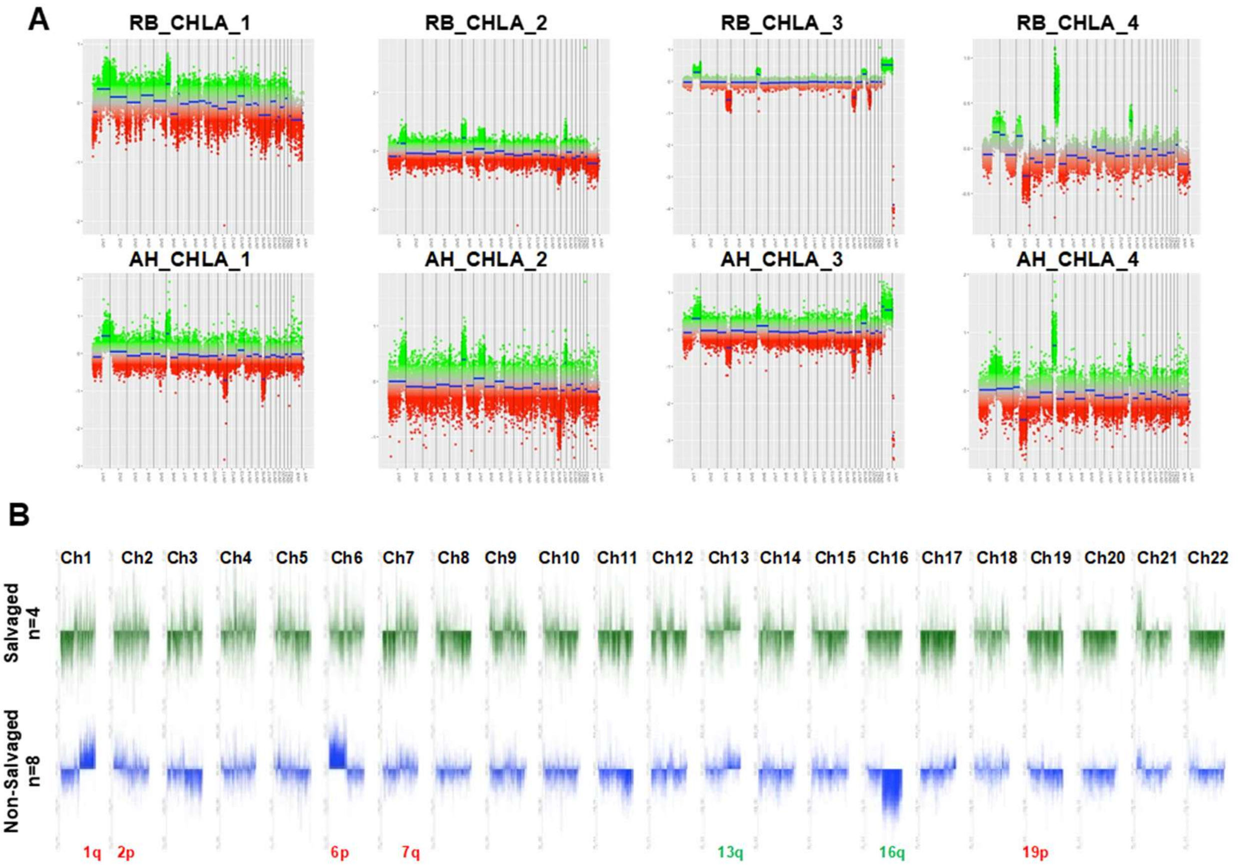

Supplementary Figure S3. Chromosomal Copy number variation (CNV) profiles from 4 eyes with AH cfDNA and corresponding paired primary tumor DNA. (A) SCNA profiles between AH and tumor pairs demonstrate the similar genomic alterations, (B) Salvaged eyes (green, n=4) had fewer copy number alterations than non-Salvaged (enucleated) eyes (blue, n=8). The gain of 1q, 2p, 6p, 7q, and 19p and loss of 13q and 16p are frequently appeared in RB.

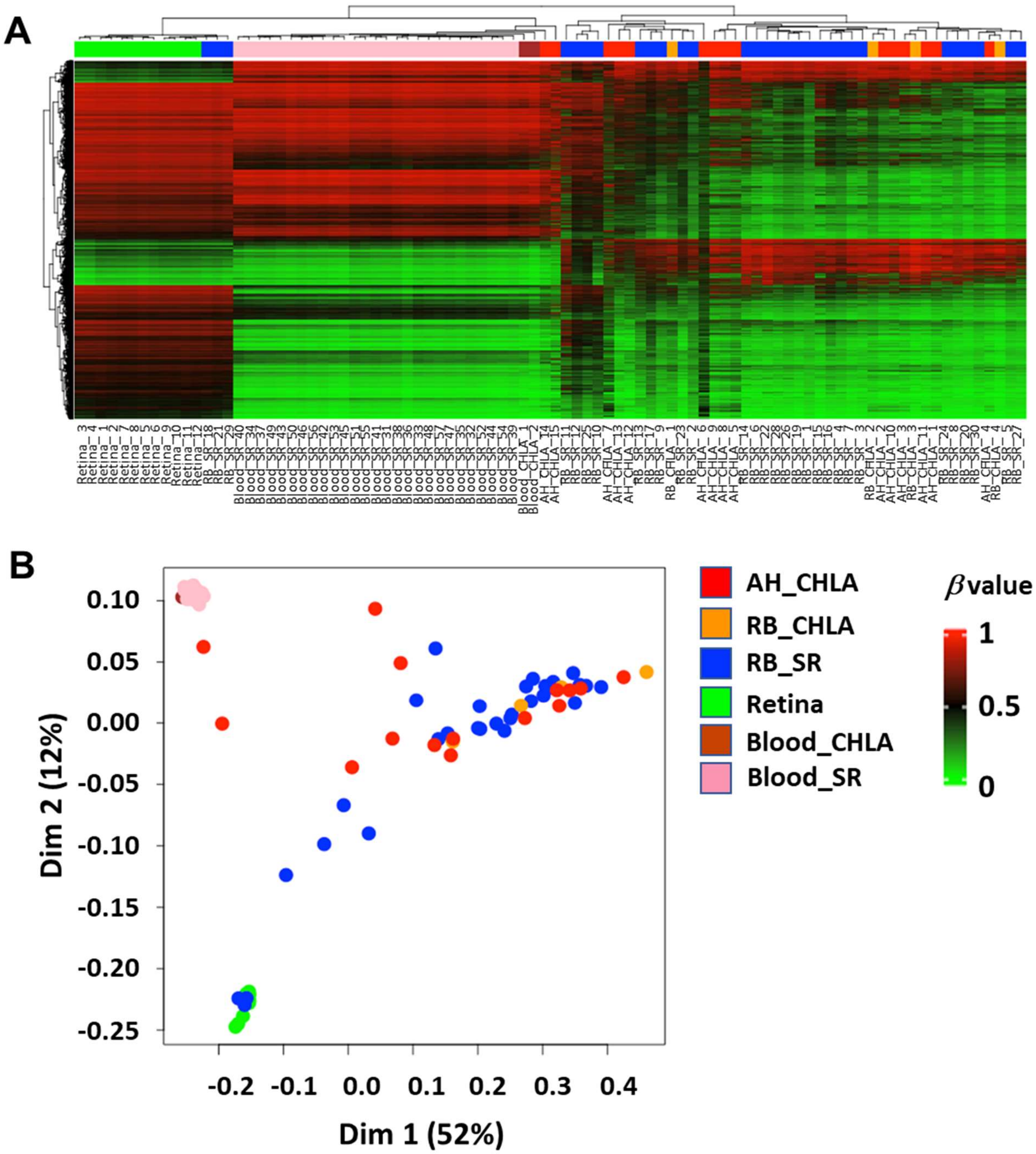

Supplementary Figure S4. Unsupervised hierarchical clustering (A) and MDS plot (B) showing the RB-specific DNA methylation profile identified in Figure 1A cannot be detected in RB patient blood samples. Sample types: AH_CHLA (red) for RB AH samples for this study, RB_CHLA (orange) for RB primary tumor samples for this study, RB_SR

(blue) for RB samples from GSE57362, Retina (green) for normal retina samples from GSE57362, Blood_CHLA (brown) for RB patient blood plasma samples in this study, and Blood_SR (pink) for RB patient white blood cell samples from GSE57362.

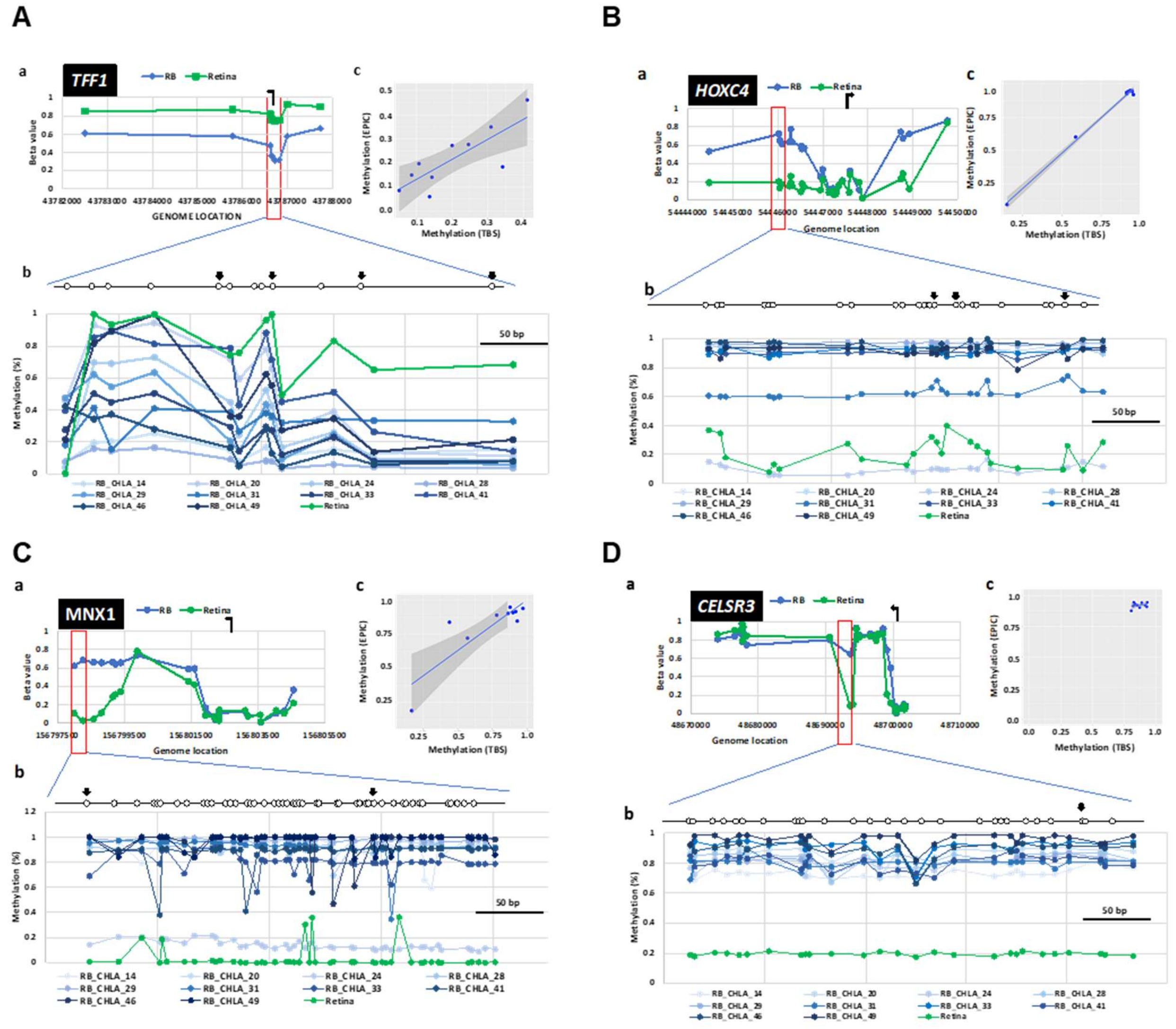

Supplementary Figure S5. DNA methylation measured by Illumina EPIC array and bisulfite targeted sequencing at the *TFF1* promoter (A), the *HOXC4* promoter (B), the *MNX1* gene body (C) and the *CELSR3* gene body (D). a) The mean DNA methylation level (βvalue) of retina and RB specimens from Figure 3A. For DNA methylation array probes at all four gene loci, the horizontal arrows indicate the transcriptional start sites; b) the mean DNA methylation status using targeted bisulfite sequencing (based on over 100 DNA molecules) for RB (n=10) and retinal (n=1) specimens. Vertical arrows indicate

the DNA methylation probe site from DNA methylation array; c) correlation of mean DNA methylation value between array (βvalue) and targeted sequencing (% methylation) for 10 RB DNA specimens. The regression lines were calculated using ggplot2 package with method “lm”. Source data are provided as a Source Data file.

Matrix	Factor name
V$ERALPHA_01	**ER group**
V$RREB1_01	**RREB-1**
V$E2F_Q6_01	**E2F related factors**
V$FPM315_01	**FPM315**
V$MAZ_Q6_01	**MAZ**
V$MUSCLEINI_B	**Muscle initiator**
V$RELA_Q6	**NF-kappaB-related factor**
V$ZFP161_04	**ZFP161**
V$EGR1_Q6	**EGR1 group**

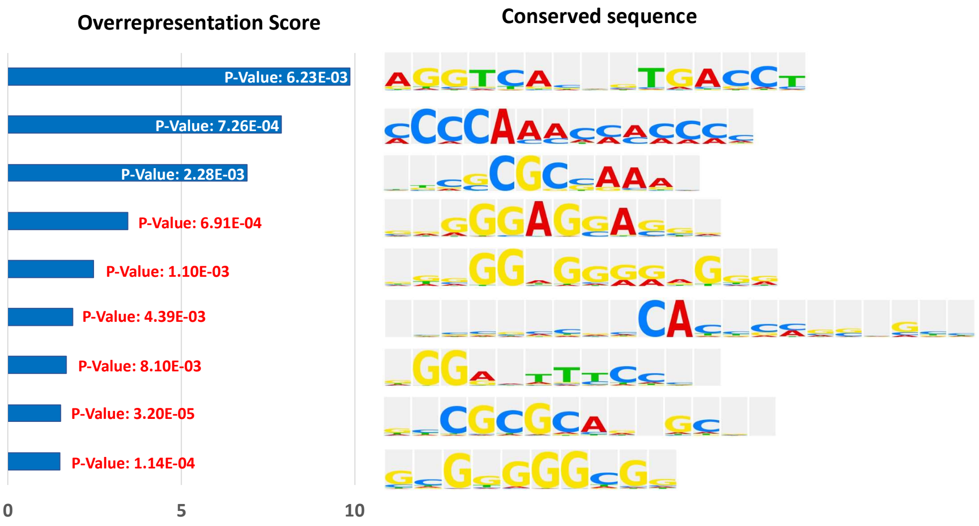

Supplementary Figure S6. Top over-represented transcription factor binding sites for methylation regulated genes (Supplementary Table 1) as determined by TRANSFAC analysis. The P values were calculated by TRANSFAC with default setting. Source data are provided as a Source Data file.
