# Supplementary material for: Characterizing DNA methylation signatures of retinoblastoma using aqueous humor liquid biopsy
Source: Nat Commun. 2022 Sep 21;13:5523. doi: 10.1038/s41467-022-33248-2 (PMC9492718; doi:10.1038/s41467-022-33248-2)
Supplement: Supplementary file 3 — Reporting Summary [file 41467_2022_33248_MOESM3_ESM.pdf]

nature portfolio

Corresponding author(s): Jesse Berry, Gangning Liang

Last updated by author(s): 08/23/2022

Reporting Summary

Nature Portfolio wishes to improve the reproducibility of the work that we publish. This form provides structure for consistency and transparency in reporting. For further information on Nature Portfolio policies, see our Editorial Policies and the Editorial Policy Checklist.

Statistics

For all statistical analyses, confirm that the following items are present in the figure legend, table legend, main text, or Methods section.

n/a	Confirmed	
☐	☒	The exact sample size (n) for each experimental group/condition, given as a discrete number and unit of measurement
☐	☒	A statement on whether measurements were taken from distinct samples or whether the same sample was measured repeatedly
☐	☒	The statistical test(s) used AND whether they are one- or two-sided<br>*Only common tests should be described solely by name; describe more complex techniques in the Methods section.*
☐	☒	A description of all covariates tested
☐	☒	A description of any assumptions or corrections, such as tests of normality and adjustment for multiple comparisons
☐	☒	A full description of the statistical parameters including central tendency (e.g. means) or other basic estimates (e.g. regression coefficient) AND variation (e.g. standard deviation) or associated estimates of uncertainty (e.g. confidence intervals)
☐	☒	For null hypothesis testing, the test statistic (e.g. F, t, r) with confidence intervals, effect sizes, degrees of freedom and P value noted<br>*Give P values as exact values whenever suitable.*
☒	☐	For Bayesian analysis, information on the choice of priors and Markov chain Monte Carlo settings
☐	☒	For hierarchical and complex designs, identification of the appropriate level for tests and full reporting of outcomes
☒	☐	Estimates of effect sizes (e.g. Cohen's d, Pearson's r), indicating how they were calculated

Our web collection on statistics for biologists contains articles on many of the points above.

Software and code

Policy information about availability of computer code

Data collection: Illumina MethylationEPIC (EPIC) DNA methylation BeadArray system was used to generate DNA methylation data.

Data analysis:
Rstudio Version 1.4.1106
R 4.1.1
minfi 1.40.1
ComplexHeatmap 2.10.0
edgeR 3.36.0
ggplot2 3.3.5
stats 4.1.1
matrixTests 0.1.9.1
circlize 0.4.14
SeSAMe 1.14.2
Ingenuity IPA 70750971
TRANSFAC 2.0
EPIC TABSAT v1.7

Code Availability
The code used for the analysis of this study is available at Github repositories (DOI: 10.5281/zenodo.7005924).

For manuscripts utilizing custom algorithms or software that are central to the research but not yet described in published literature, software must be made available to editors and reviewers. We strongly encourage code deposition in a community repository (e.g. GitHub). See the Nature Portfolio guidelines for submitting code & software for further information.

Data

Policy information about availability of data

All manuscripts must include a data availability statement. This statement should provide the following information, where applicable:

- Accession codes, unique identifiers, or web links for publicly available datasets
- A description of any restrictions on data availability
- For clinical datasets or third party data, please ensure that the statement adheres to our policy

The raw DNA methylation datasets for this study are available at the Gene Expression Omnibus GSE208055 (https://www.ncbi.nlm.nih.gov/geo/query/acc.cgi?acc=GSE208055) and GSE211508 (https://www.ncbi.nlm.nih.gov/geo/query/acc.cgi?acc=GSE211508).
TRANSFAC dataset (2.0, genexplain, Germany) was used for transcription factor binding sites prediction.
The public DNA methylation data of normal retina and RB tumors used in this study are available in GEO database under accession codes GSE57362 and GSE58783.
The processed expression array data (GSE125903 and GSE111168) were downloaded from BaseSpace correlation engine.
The remaining data are available within the Article, Supplementary Information, or Source Data File. Additional data in inquiry can be addressed to the Lead Contact: jesse.berry@med.usc.edu.
Source data are provided with this paper.

Field-specific reporting

Please select the one below that is the best fit for your research. If you are not sure, read the appropriate sections before making your selection.

- [x] Life sciences
- [ ] Behavioural & social sciences
- [ ] Ecological, evolutionary & environmental sciences

For a reference copy of the document with all sections, see nature.com/documents/nr-reporting-summary-flat.pdf

Life sciences study design

All studies must disclose on these points even when the disclosure is negative.

Sample size	We included a first series of 15 aqueous humors samples collected from 13 retinoblastoma (RB) eyes for genome-wide methylation profiling. Among these 15 aqueous humor samples, 9 of them were collected from RB eyes still undergoing active treatment, thus makes them the first cohort of in vivo AH samples undergoing methylation profiling. We used another cohort of 11 RB primary tumors and 1 healthy retina DNA for DNA methylation validation. No sample size calculation was performed.
Data exclusions	No data were excluded.
Replication	No replication due to limited amount of clinical samples.
Randomization	This was a retrospective study. The work shows DNA methylation of cfDNA from aqueous humour is altered in retinoblastoma patients and can be used to identify the molecular subtypes and potentially predict treatment response. Treatment decisions were made independently of this study, so randomization is not applicable.
Blinding	This was a retrospective study of rare disease. Treatment decisions were made independently of this study. No statistical method was used to predetermine sample size. Blindness of clinical study does not apply to our study.

Reporting for specific materials, systems and methods

We require information from authors about some types of materials, experimental systems and methods used in many studies. Here, indicate whether each material, system or method listed is relevant to your study. If you are not sure if a list item applies to your research, read the appropriate section before selecting a response.

Materials & experimental systems

n/a	Involved in the study	
x		Antibodies
	x	Eukaryotic cell lines
x		Palaeontology and archaeology
x		Animals and other organisms
	x	Human research participants
	x	Clinical data
x		Dual use research of concern

Methods

n/a	Involved in the study	
x		ChIP-seq
x		Flow cytometry
x		MRI-based neuroimaging

Eukaryotic cell lines

Policy information about cell lines

Cell line source(s)	ATCC
Authentication	ATCC Cell Line Authentication Service
Mycoplasma contamination	Cell line was detected negative of mycoplasma.
Commonly misidentified lines (See ICLAC register)	*Name any commonly misidentified cell lines used in the study and provide a rationale for their use.*

Human research participants

Policy information about studies involving human research participants

Population characteristics	4 Tumors and 15 AH specimens were collected from 12 patients with retinoblastoma at Children's Hospital Los Angeles (CHLA). Among these 12 patients, 6 of them are females and 6 are males, age ranging from 2-35 months (17.6±9.48 months) which represents RB disease characteristics.<br><br>For all participants, treatments were performed per routine CHLA protocol. Treatment decisions were made independently of this study.
Recruitment	Patients were randomly recruited from Children's Hospital Los Angeles retinoblastoma clinics. 96% RB patients of CHLA consented to participate into aqueous humor study.
Ethics oversight	This research is conducted under Institutional Review Board approval (CHLA-17-00248). Written informed consent is obtained from the parents of all participants prior to inclusion in the study.

Note that full information on the approval of the study protocol must also be provided in the manuscript.

Clinical data

Policy information about clinical studies

All manuscripts should comply with the ICMJEguidelines for publication of clinical research and a completedCONSORT checklist must be included with all submissions.

Clinical trial registration	Not applicable.
Study protocol	Not applicable.
Data collection	Per routine CHLA protocol.
Outcomes	Three type of outcomes: Salvaged eyes, Primary enucleation for eyes with advanced disease so eucleated without any medical intervention; secondary enucleation for eyes failed active treatment with eyes surgically removed.
