# Supplementary material for: Characterizing DNA methylation signatures of retinoblastoma using aqueous humor liquid biopsy
Source: Nat Commun. 2022 Sep 21;13:5523. doi: 10.1038/s41467-022-33248-2 (PMC9492718; doi:10.1038/s41467-022-33248-2)
Supplement: Supplementary file 4 — description of additional supplementary files [file 41467_2022_33248_MOESM4_ESM.pdf]

Description of Additional Supplementary Files

File Name: Supplementary Data 1 (xlsx)
Description: The list of differentially expressed genes which are associated with DNA methylation changes between retinoblastoma and healthy retina.

File Name: Supplementary Data 2 (xlsx)
Description: The differentially methylated illumine EPIC array probes between cluster A and cluster B.

File Name: Supplementary Data 3 (xlsx)
Description: The RB samples used for validation of the illumine EPIC array data with target bisulfite sequencing.

File Name: Supplementary Figures and Figure Legends (PDF)
Description: 6 supplementary figures and corresponding figure legends
